# Radical Roles for RAGE in the Pathogenesis of Oxidative Stress in Cardiovascular Diseases and Beyond

**DOI:** 10.3390/ijms141019891

**Published:** 2013-10-01

**Authors:** Gurdip Daffu, Carmen Hurtado del Pozo, Karen M. O’Shea, Radha Ananthakrishnan, Ravichandran Ramasamy, Ann Marie Schmidt

**Affiliations:** Diabetes Research Program, Division of Endocrinology, Department of Medicine, New York University Langone Medical Center, 550 First Avenue, Smilow 901C, New York, NY 10016, USA

**Keywords:** receptor for advanced glycation endproduct, non-enzymatic glycation, inflammation, redox signaling

## Abstract

Oxidative stress is a central mechanism by which the receptor for advanced glycation endproducts (RAGE) mediates its pathological effects. Multiple experimental inquiries in RAGE-expressing cultured cells have demonstrated that ligand-RAGE interaction mediates generation of reactive oxygen species (ROS) and consequent downstream signal transduction and regulation of gene expression. The primary mechanism by which RAGE generates oxidative stress is via activation of NADPH oxidase; amplification mechanisms in the mitochondria may further drive ROS production. Recent studies indicating that the cytoplasmic domain of RAGE binds to the formin mDia1 provide further support for the critical roles of this pathway in oxidative stress; mDia1 was required for activation of rac1 and NADPH oxidase in primary murine aortic smooth muscle cells treated with RAGE ligand S100B. *In vivo*, in multiple distinct disease models in animals, RAGE action generates oxidative stress and modulates cellular/tissue fate in range of disorders, such as in myocardial ischemia, atherosclerosis, and aneurysm formation. Blockade or genetic deletion of RAGE was shown to be protective in these settings. Indeed, beyond cardiovascular disease, evidence is accruing in human subjects linking levels of RAGE ligands and soluble RAGE to oxidative stress in disorders such as doxorubicin toxicity, acetaminophen toxicity, neurodegeneration, hyperlipidemia, diabetes, preeclampsia, rheumatoid arthritis and pulmonary fibrosis. Blockade of RAGE signal transduction may be a key strategy for the prevention of the deleterious consequences of oxidative stress, particularly in chronic disease.

## 1. RAGE—A Molecule of the Immunoglobulin Superfamily and Links to Oxidative Stress

RAGE was first identified on account of its ability to bind advanced glycation endproducts (AGEs), the products of nonenzymatic glycation and oxidation of proteins and lipids [1]. RAGE is expressed in a wide array of cell types, such as vascular cells, inflammatory cells, glomerular epithelial cells (podocytes), neurons of the central and peripheral nervous systems, cardiomyocytes, retinal Muller ganglion cells and alveolar pneumocytes [2]. In homeostasis, RAGE is usually expressed at low levels in adult, non-diseased tissues; in settings such as cardiovascular disease, diabetes, inflammation, tumors and neurodegeneration, expression of RAGE is higher than that observed in control, non-diseased animal models or human subjects [3]. The key to the biology of RAGE is its ability to bind to and transduce the molecular effects of multiple ligands. In addition to AGEs, RAGE transduces the signals of distinct ligands, such as multiple members of the S100/calgranulin family [4], high mobility group box 1 (HMGB1) [5], amyloid beta-peptide and beta-sheet fibrils [6], lysophosphatidic acid (LPA) [7], Mac1 [8], and C1q [9].

The biological impact of RAGE is diverse and depends on the cell type, acuteness *vs*. chronicity of stimulation and interactions with surrounding cell types *in vivo*, such those released in an autocrine and/or paracrine manner which may modulate RAGE action. We propose that RAGE ligands may be sequentially induced and released depending on the nature of the cellular stress. For example, our observation that endothelial cells release AGEs within minutes of exposure to hypoxia suggests that RAGE ligands may amplify and perpetuate acute and chronic stresses within the tissues [10]. We and others have shown that RAGE activates a diverse array of signaling cascades with significant and wide-ranging impact on gene expression profiles characterized by inflammation, fibrosis and cell fate, as examples.

In the context of natural aging and diabetes, it is speculated that the development, perpetuation and unremitting nature of reactive oxidant species (ROS) generation directly contributes to the complications of these two key states [11,12]. Compelling evidence highlights pathogenic roles for RAGE in both diabetes and aging. In this review, we will focus on the evidence linking ligand-RAGE interaction to oxidative stress and its consequences.

## 2. RAGE and Oxidative Stress—First Evidence

We first described roles for AGE-RAGE interaction in endothelial cells. AGEs, either those prepared *in vitro* or via immunoisolation from diabetic plasma, resulted in generation of thiobarbituric acid reactive substances (TBARS) and activation of oxidant stress-sensitive NF-κB. Roles for RAGE and oxidative stress in the biological impact of AGEs were evident by blockade of these effects by pre-treatment of the endothelial cells with blocking antibodies to RAGE or anti-oxidants [13]. *In vivo* studies demonstrated that infusion of AGEs into animals resulted in increased oxidative stress as evidenced by increased levels of malondialdehyde and TBARS in the vasculature and tissues, activation of NF-κB and upregulation of heme oxygenase 1 mRNA. Pre-treatment of the animals with anti-RAGE antibodies or antioxidants blocked these effects of infused AGEs [13]. A key question from this work, however, was by what means AGEs generated oxidative stress via RAGE.

Subsequent studies detailed key roles for AGEs in generation of oxidative stress via activation of NADPH oxidase. Specifically, Wautier and colleagues showed that incubation of AGEs on the surface of diabetic red blood cells or the specific AGE, carboxy methyl lysine (CML) AGE with endothelial cells resulted in generation of hydrogen peroxide, upregulation of vascular cell adhesion molecule 1 (VCAM-1) and generation of tissue factor. Treatment of the endothelial cells with diphenyliodonium (DPI), but not by inhibitors of nitric oxide, blocked these effects of AGEs. The key role of NADPH oxidase was shown in these effects of AGEs was shown for the first time by the failure of macrophages retrieved from mice deficient in gp91phox but not wild-type (WT) mice to upregulate tissue factor upon incubation with AGEs [14].

Further experiments suggested that AGE-dependent activation of oxidative stress via RAGE in renal mesangial cells facilitated the generation of mitochondrial superoxide. Infusion of AGEs into non-diabetic rodents resulted in increased cytosolic ROS, followed subsequently by increase mitochondrial permeability transition and deficiency of mitochondrial complex I. These effects were even further pronounced in diabetic rodents [15]. Supportive of the roles of cytosolic ROS generated from AGE-RAGE interaction in the induction of mitochondrial oxidative stress were the findings that reduction of cytosolic ROS generation (using apocynin) or lowering AGEs (using alagebrium, an AGE cross-link breaker agent) reduced mitochondrial superoxide generation. Further, in mice devoid of RAGE, diabetes-induced increases in mitochondrial superoxide in the renal cortex were reduced [15]. These data suggest that AGE-RAGE interaction may initiate generation of ROS, largely via cytosolic NADPH oxidase-dependent mechanisms, but that in certain conditions and cell types, AGE-RAGE may contribute to downstream amplification of oxidative stress via the induction of mitochondrial superoxide.

These studies led therefore to the question of the extent to which RAGE ligands activated oxidative stress in cell types other than endothelial cells, macrophages or renal mesangial cells. In the section to follow, we detail the evidence that RAGE ligands activate oxidative stress in a broad array of cell types, thereby implicating this receptor pathway in fundamental mechanisms of oxidative stress and tissue-specific consequences.

## 3. RAGE and Oxidative Stress: Evidence in Multiple Cell Types

RAGE is expressed on diverse cell types. A common mechanism by which the ligands of RAGE may modulate the biological properties of these cells is via oxidative stress. As discussed above, RAGE ligand action in endothelial cells contributes to the generation of oxidative stress. In the sections to follow, we illustrate examples of RAGE-dependent oxidative stress in cardiovascular cells and beyond.

### 3.1. Cardiac Endothelial Cells

In primary rat cardiac microvascular endothelial cells, incubation with AGE prior to simulated ischemia and reperfusion resulted in increased release of lactic dehydrogenase (LDH) and caspase activity. The underlying mechanisms were linked to generation of oxidative stress, as evidenced by increased expression of iNOS, total nitric oxide production, formation of superoxide and peroxynitrite and nitrative inactivation of thioredoxin 1 (Trx-1), the latter essential for cellular protection [16]. The adverse effects of AGE pretreatment prior to simulated ischemia/reperfusion were prevented by blockade of RAGE signaling. These findings underscore potential mechanisms by which diabetes or aging (in which AGEs accumulate) may predispose the cardiovasculature to increased vulnerability to damage initiated by ischemia and reperfusion.

### 3.2. Vascular Smooth Muscle Cells (SMCs)

In SMCs, RAGE ligand S100B resulted in increased production of superoxide, migration and expression of MCP-1 and IL6 in a manner dependent on S100B-induced activation of Src kinase and ERK signaling, together with activation of the transcription factors NF-κB and STAT3 [17].

In other studies, Hofmann Bowman and colleagues showed that transgenic expression of RAGE ligand S100A12 in SMCs mediated oxidative stress in pathways linked to vascular calcification. They showed that inhibition of NADPH oxidase blocked the effects of S100A12 on osteogenic gene programming and calcification mechanisms [18]. Specifically, they linked these findings to a direct interaction between Nox1 and S100A12.

### 3.3. Inflammatory Cells

As discussed above, RAGE is expressed on monocytes/macrophages. Macrophages retrieved from mice deficient in gp91phox that failed to respond to AGEs solidified roles for NADPH oxidase in the mechanisms linking RAGE to oxidative stress [14]. Other inflammatory cell types express RAGE, suggesting that oxidative stress is linked to the mechanisms of RAGE ligand-mediated perturbation.

Neutrophils play prominent roles in infection, inflammation and diabetes. Omori and colleagues demonstrated that neutrophils retrieved from diabetic subjects displayed basally increased translocation of p47phox, a key component of activation of NADPH oxidase, to the cell membrane, in parallel with enhanced preassembly with p22phox. These findings were modeled in cultured HL60 cells in which it was shown that exposure of these cells to high glucose or to RAGE ligand S100B resulted in translocation of p47phox to the cell membrane in a manner dependent on ERK signal transduction and RAGE [19].

In mast cells, which express RAGE, exposure of these cells to galectin-3 results in release of superoxide with consequent opening of the mitochondrial transition pore and caspase-dependent apoptosis. Consistent with key roles for RAGE, these effects of galectin-3 were blocked by pre-treatment of the cells with antibodies to RAGE or bongkrekic acid, the latter an antagonist of the mitochondrial transition pore [20].

### 3.4. Endothelial Progenitor Cells (EPCs)

In diabetes and aging [21,22], repair mechanisms are thwarted in concert with increased cellular stress and damage. In bone marrow derived EPCs, exposure to LPS-free *C*-reactive protein (CRP) (at physiologically-relevant concentrations) induced ROS generation, upregulation of RAGE expression, reduced antioxidant defenses and induction of EPC apoptosis. These processes were prevented by pre-treatment with anti-RAGE IgG [23]. In other studies, incubation of EPCs with AGEs resulted in induction of ROS production, reduced anti-oxidant defenses, inhibition of EPC proliferation, migration and adhesion, and induction of EPC apoptosis. These effects were prevented by blockade of RAGE [24]. In summary, the authors surmised that AGEs increased the vulnerability of EPCs to exogenous stresses and that RAGE is a key principal mediator of oxidative and cell death stresses in these cells.

### 3.5. Cells of the Nervous System

Amyloid beta-peptide (Aβ) and beta sheet fibrils are specific ligands of RAGE. This ligand-RAGE interaction plays key roles in mechanisms linked to neuronal stress in Alzheimer’s disease. In cultured neuronal cells, Aβ-RAGE interaction resulted in generation of TBARS and activation of NF-κB, processes which were dependent on oxidative stress as demonstrated by blockade of these effects by *N*-acetyl cysteine [6]. Furthermore, Aβ-RAGE mediated oxidative stress in neuronal cells results in upregulation of macrophage colony stimulating factor (MCS-F) through an NF-κB-dependent pathway [25]. Roles for oxidative stress were uncovered by the blocking effects of *N*-acetyl cysteine in this culture model. Notably, the upregulation of MCS-F was shown to stimulate microglial cell migration, proliferation, and survival, factors contributing to neuroinflammation in Alzheimer brain.

In cultured human NT2 neurons, exposure of these cells to increasing concentrations of the specific AGE, pentosidine, resulted in generation of ROS and apoptotic cell death. In parallel, expression of RAGE rose in a manner dependent of the concentration of AGEs [26]. The mechanistic role for PKC-delta in pentosidine-mediated generation of ROS and upregulation was demonstrated by blockade of these effects by pretreatment of the neurons with rottlerin, an inhibitor of PKC-delta. Furthermore, pre-treatment of the neurons with the antioxidant, Vitamin E, also prevented generation of ROS and RAGE upregulation, thus suggesting that a key mechanism by which pentosidine initiated these effects was via generation of ROS.

In cultured astrocytes, treatment with AGE-bovine serum albumin (BSA) induced increased production of tumor necrosis factor (TNF)-α and Interleukin 1β, in parallel with increased oxidative stress, as measured by reduced astrocyte glutathione (GSH) levels and increased nitric oxide (NO) release [27]. Although the direct link to RAGE signaling was not demonstrated, the authors did show that RAGE was expressed by astrocytes.

In other studies, RAGE was shown to be expressed by primary sensory neurons, thereby suggesting potential links of the RAGE pathway to peripheral nervous system disorders. Vincent and colleagues showed that incubation of primary sensory neurons with the RAGE ligand S100 resulted in generation of oxidative stress and consequent increased activity of phosphatidyl inositol-3-kinase and activation of apoptotic mechanisms [28]. These processes were prevented by pretreatment of the neurons with the anti-oxidant α lipoic acid.

Intriguingly, in other cells of the nervous system, specifically oligodendrocytes, oxidative stress modulated shedding of RAGE from the cell surface. Qin and colleagues showed that incubation of neonatal rat oligodendrocytes with low doses of hydrogen peroxide (100 μM) resulted in shedding of RAGE from the cell surface into the culture medium, in parallel with restoration of homeostatic levels of RAGE on the cell surface. In contrast, when the cells were exposed to high doses of hydrogen peroxide (>200 μM), the cells were shown to undergo cell death [29]. Of note, both antioxidants and metalloprotease inhibitors blocked these effects in oligodendrocytes. These findings may have implications for diseases in which demyelination predominates, such as in multiple sclerosis and Batten’s disease (neuronal ceroid-lipofuscinosis).

### 3.6. Osteoblast Cells

AGEs have been shown to impart adverse consequences on osteoblasts; this bears implications for aging-associated bone disorders and diabetic complications. Schurman and colleagues studied two osteoblast cell lines, UMR106 and MC3T3E1 cells, and showed that incubation with AGEs stimulated oxidative stress, activation of caspase 3 activity and inhibition of alkaline phosphatase activity [30]. In these cells, AGEs resulted in upregulation of RAGE protein. Of note, the authors showed that incubation of osteoblast cultured cells with metformin prevented the adverse consequences of AGE treatment.

### 3.7. Renal Cells

In addition to the roles for AGE-stimulated mitochondrial oxidative stress post-activation of cytosolic ROS in mesangial cells as discussed above, distinct studies in cultured mesangial cells highlighted roles for pigment epithelium derived factor (PEDF) in protection against AGE-induced oxidative and cellular damage. Ide and colleagues incubated renal mesangial cells with AGEs and identified increased oxidative stress, activation of NF-κB and upregulation of MCP-1, VCAM-1 and PAI-1, all in a manner suppressed by treatment with PEDF or blocking antibodies to RAGE [31].

In renal interstitial fibroblasts (NRK-49F cells), incubation with AGEs increased ROS and via ERK signaling induced cellular mitogenesis in a manner suppressed by antioxidants (*N*-acetyl cysteine) and reduction of RAGE expression using RAGE short hairpin RNA [32].

### 3.8. Pancreatic β Cells

The effects of RAGE ligands on pancreatic β cells were studied in cultured rat INS-1 (insulinoma) cells. RAGE was found to be expressed by these cells and incubation with two distinct RAGE ligands, S100B or HMGB1, induced intracellular oxidative stress and cellular apoptosis. Consistent with key mechanistic roles for oxidative stress in RAGE ligand-mediated cell stress, pre-treatment of the cells with antioxidants or an NADPH oxidase inhibitor blocked these adverse effects on pancreatic β cells [33]. These findings might suggest that in pre-diabetes syndromes or in established diabetes, the increased production of AGEs (stimulated likely by hyperglycemia) might contribute to further derangement of glucose metabolism via direct attack on pancreatic insulin-producing cells.

In the section to follow, we discuss examples in which RAGE was directly linked to oxidative stress *in vivo*.

## 4. RAGE and Oxidative Stress: Evidence from *in Vivo* Models

*In vivo* model systems have provided support for roles for RAGE in oxidative stress in various conditions and tissues.

### 4.1. Cardiovascular System

Studies have addressed the role of RAGE and oxidative stress in the context of such disorders as atherosclerosis, aneurysm formation and ischemia-reperfusion injury.

Sun and colleagues tested the role of RAGE in atherosclerosis using mice deficient in the low density lipoprotein receptor (LDLR) and fed a western-type diet (0.15% cholesterol). Compared to RAGE expressing LDLR null mice, those mice devoid of RAGE displayed decreased atherosclerosis in parallel with reduced levels of vascular oxidative stress, as measured by detection of superoxide by dihydroethidium [34].

In a distinct model, Hofmann Bowman and colleagues demonstrated that SMC specific overexpression of RAGE ligand S100A12 resulted in the development of aneurysm formation in the aorta. In parallel, evidence for increased oxidative stress, as measured by myeloperoxidase activity, was significantly higher in the transgenic *vs*. wild-type mouse vasculature [35]. In primary cultures of SMCs from the transgenic and wild-type mice, increased oxidative stress was evident in these cells by increased staining for MitoSox (marker of mitochondrial oxidative stress), 8-hydroxy-2-deoxyguanosine and mitochondrial oxidative stress [35]. As RAGE is the principal receptor for S100A12, it is highly plausible that these effects *in vivo* and in SMCs examined *ex vivo* were mediated via RAGE, but these concepts regarding potential roles for RAGE were not directly tested in this study.

Tikellis and colleagues tested the role of RAGE and oxidative stress *in vivo* in a model of high fat feeding. They showed that RAGE-deficient mice fed a Western-type diet, composed of 21% fat, displayed reduced markers of oxidative stress, including NADH- and NADPH-dependent mitochondrial superoxide production, inflammation and hypertrophy in the heart compared to the wild-type animals. Treatment with the AGE inhibitor, alagebrium, further reduced oxidative stress and inflammation in both RAGE null and the wild-type mice [36].

Oxidative stress contributes critically to the pathogenesis of ischemia-reperfusion injury. Bucciarelli and colleagues showed that cardiac ischemia-reperfusion led to the upregulation of RAGE, inducible nitric oxide synthase expression and increased levels of nitric oxide, cyclic guanosine monophosphate (cGMP) and nitrotyrosine in both rat and mouse hearts. This was prevented by administration of soluble RAGE or by genetic deletion of RAGE in both non-diabetic and diabetic animals [37,38].

A consequence of ischemia-reperfusion is mitochondrial oxidative stress and the opening of the mitochondrial transition pore and cytochrome c release, harbingers to the activation of cell death apoptotic pathways [39,40]. In a murine model of ligation and reperfusion of the left anterior descending coronary artery, genetic deletion of RAGE or administration of soluble RAGE significantly reduced release of cytochrome c and resulted in significant attenuation of infarct volume and the loss of cardiac function post-infarct. Signal transduction studies underscored key roles for JNK and STAT signaling in these oxidative stress–cell death mechanisms [41].

In other studies, Tsoporis and colleagues induced myocardial infarction in rats and showed that this resulted in upregulation of RAGE expression in cardiomyocytes and release of RAGE ligand S100B, in parallel with myocyte apoptosis. Direct interactions between S100B and RAGE were illustrated by co-immunoprecipitation experiments. In rat neonatal cardiomyocyte cultures, S100 caused increased release of cytochrome c and cardiomyocyte apoptosis [42].

### 4.2. Renal System

In the kidney, roles for RAGE in oxidative stress in distinct pathophysiologic settings including diabetes-associated nephropathy, adriamycin (doxorubicin) toxicity and obesity associated renal dysfunction have been demonstrated.

Inagi and colleagues developed a triple transgenic mouse model of diabetes characterized by transgene-mediated expression of RAGE, iNOS and megsin. In the kidneys of these mice, significant acceleration of renal indices of nephropathy was shown in parallel with evidence of increased oxidative stress in triple transgenic mice *vs*. the controls, including increased accumulation of the AGEs pentosidine and CML AGE, and increased urinary levels of 8-oxo-2′-deoxyguanosine (8-OHdG) [43].

Roles for RAGE in induction of renal dysfunction in obesity were demonstrated. When mice were fed a high fat diet and developed obesity, renal levels of mitochondrial superoxide and cytosolic superoxide were increased in the renal cortices of the animals compared to control lean mice. However, in RAGE null mice fed the high fat diet, levels of these two measures of oxidative stress were significantly attenuated. Furthermore, increased AGE CML noted in the kidneys of obese wild-type mice was significantly lower in obese RAGE null mice [44]. These data strongly suggested that obesity-associated renal oxidative stress was in mediated at least in part via AGE-RAGE.

Further compelling evidence for roles for RAGE in renal associated oxidative stress emerged from experiments in which the nephrotoxin adriamycin (doxorubicin) was administered to wild-type or RAGE null mice (in the Balb/c background). RAGE null mice were strikingly protected against the adverse effects of adriamycin damage on podocyte effacement and glomerular sclerosis [45]. A key mediator of adriamycin toxicity is postulated to be the induction of oxidative stress. Furthermore, exposure of wild-type mice to adriamycin resulted in generation of AGEs in the renal cortex. Consistent with roles for RAGE in oxidative stress damage in this model, RAGE null mice treated with adriamycin displayed marked reduction in renal levels of NADPH oxidase activity, malondialdehyde, iNOS transcripts, total nitrite and nitrate, and nitrotyrosine levels. Similar results were obtained when wild-type BALB/c mice were treated with adriamycin and soluble RAGE (sRAGE) [45]. In cultured podocytes, RAGE ligand (S100B) stimulation resulted in activation of NADPH oxidase in a manner dependent on ERK signaling. These data provided compelling evidence linking RAGE to oxidative stress and renal fibrosis consequent to injection with adriamycin.

### 4.3. Central Nervous System

To link RAGE and oxidative stress in the central nervous system, rats were treated with high doses of Vitamin A and the cerebral cortices were removed after treatment. At doses of Vitamin A greater than or equal to 2500 IU/kg/day, increased measures of oxidative stress were noted in the brain, as evidenced by higher levels of superoxide anion, carbonylation and 3-nitrotyrosine. In parallel with increased oxidative stress, RAGE protein levels (as assessed by Western blot) were significantly higher in the brain tissue in rats fed greater than or equal to 4500 IU/kg/day Vitamin A [46]. Although these data did not establish a causal effect between RAGE expression and generation of oxidative stress in rats fed high doses of Vitamin A nor did they address the pattern of expression of RAGE ligands in Vitamin A feeding, they nevertheless supported that a relationship between the RAGE pathway and oxidative stress was mechanistically plausible *in vivo*.

### 4.4. Liver

One consequence of chronic oxidative stress is the generation of AGEs and this is evidenced by the fact that oxidative stress induces hepatic protein glycation. Kuhla and colleagues showed that mice deficient in Uncoupling Protein 2 (UCP2) demonstrated increased oxidative stress (as determined by lower ratio of GSH to GSSG) [47]. In parallel, increased oxidative stress was linked to reduced activity of glyoxalase 1 (Glo1); Glo1 is a chief enzyme responsible for the detoxification of major AGE precursors methylglyoxal and glyoxal [48]. The authors showed that AGE and RAGE levels in the livers of UCP2 null mice were increased and that levels of the soluble RAGE decoy, soluble RAGE (sRAGE), were greatly reduced in these animals compared to the wild-type control mice [47]. When liver injury was induced in these animals, administration of recombinant soluble RAGE exerted protection. The authors deduced that sRAGE’s effects were mediated, at least in part, by the binding of RAGE ligands, as AGE levels were indeed reduced in the sRAGE-treated UCP2 null mice.

Further evidence for RAGE-mediated oxidative stress in the liver was evident in a murine model of acetaminophen toxicity. Administration of soluble RAGE to mice receiving toxic doses of this agent resulted in decreased levels of nitrotyrosine adducts in liver tissue together with higher levels of glutathione [49]. In parallel with reduced measures of oxidative stress, soluble RAGE-treated mice displayed improved survival compared with those animals treated with vehicle [49].

These data further buttress the concept that AGE-RAGE may incite a vicious cycle of oxidative stress and the induction of mechanisms that sustain and amplify ROS generation as well as inflammatory signals, mediated by the release of pro-inflammatory RAGE ligands, namely S100/calgranulins and HMGB1, in chronic stress. In the section to follow, we discuss novel insights into mechanisms by which RAGE signal transduction facilitates the generation of oxidative stress.

## 5. RAGE Signaling and Novel Roles for RAGE Cytoplasmic Domain Binding Partner, mDia1, in Oxidative Stress

The ligands of RAGE transduce their signals through this receptor by activation of a range of signaling pathways; the precise signaling pathways involved in RAGE action appear to be determined based on the nature of the cellular stress and the cell type. *In vivo*, undoubtedly, the signals emitted by surrounding cells likely further dictate the signaling pathways activated upon RAGE ligand stimulation. We discovered that the cytoplasmic domain of RAGE binds to the formin molecule mDia1 and that mDia1 is required for RAGE ligands to activate cell signaling responses in multiple cell types such as transformed cells, smooth muscle cells, and monocytes/macrophages [50–52]. Formins such as mDia1 (diaphanous1) are actin binding molecules that contribute to signal transduction mechanisms in part via effecting Rho GTPase signals [53]. Hence, given the important role of Rac1, one of the Rho GTPases, as a component of activated NADPH oxidase, we tested the hypothesis that mDia1 was essential both for RAGE ligand-mediated generation of oxidative stress and for RAGE ligand-mediated signal transduction and cellular migration.

The role of mDia1 was studied in experiments in SMCs and in a murine model of arterial injury induced by endothelial denudation of the femoral artery [52]. In this *in vivo* model, oxidative stress induced by the injury was significantly attenuated in both RAGE null and mDia1 null mice compared to wild-type control animals. The key role of mDia1 in this process was underscored by experiments which revealed the following: first, deletion of mDia1 *in vivo* reduced NADPH oxidase in the injured vessel; second, *in vitro*, exposure of SMCs retrieved from the aorta of mice to RAGE ligand S100B increased ROS production; this was prevented in SMCs retrieved from RAGE null or mDia1 null mice; third, in wild-type SMCs, treatment with S100B resulted in increased membrane translocation of p47phox and Nox1 with no effect on Nox4; this was prevented in RAGE null or mDia1 null SMCs; fourth, activation of Rac1, a key component of NADPH oxidase, was noted in wild-type but not RAGE null or mDia1 null SMCs treated with S100B; fifth, blockade of Rac1 with dominant negative Rac1 or blockade of Nox1 with siRNAs targeting Nox1 reduced S100B-mediated generation of ROS, suppressed activation of serine 9 GSK-3β; and sixth, treatment of wild-type SMCs with dominant negative Rac1 blocked S100B-mediated migration [52]. These data revealed for the first time that mDia1 was a critical regulator of oxidative stress generation and SMC migration in response to RAGE ligands. Further experimentation is underway to determine if and by what mechanisms mDia1 might be linked to activation of NF-κB.

Finally, in the section to follow, we address evidence linking ligand-RAGE actions to oxidative stress mechanisms in human subjects.

## 6. AGE-RAGE and Oxidative Stress: Evidence from Human Subjects

Evidence is emerging from the study of human tissues and plasma to link ligand-RAGE activity to oxidative stress in human subjects bearing a range of pathological conditions—from central nervous disorders to rheumatoid arthritis. Although seemingly correlative, these findings nevertheless place ligand-RAGE and oxidative stress together *in vivo* in human subjects and suggest mechanistic roles for this pathway in oxidative stress damage as well as the possibility that ligand-RAGE might provide novel biomarkers into these pathobiological mechanisms.

### 6.1. Cardiometabolic and Renal Disease

Subjects with renal failure undergoing hemodialysis treatment are highly susceptible to acceleration of cardiovascular disease, irrespective of the etiology of loss of renal function. The link between inflammation and oxidative stress in this disorder is therefore of considerable interest. Rodriguez-Ayala and colleagues examined 7 subjects on hemodialysis with high-grade inflammation (*C*-reactive protein levels > 10 mg/L) and 11 subjects with low-grade inflammation (*C-*reactive protein levels < 10 mg/L) for at least a six month period. The authors found that the subjects with high-grade inflammation displayed higher levels of IL-6, myeloperoxidase activity and advanced oxidation protein products (AOPPs) *vs*. the low-grade inflammation subjects [54]. In the subject group with high-grade inflammation, stimulation of their monocytes with CML-human serum albumin resulted in a rapid increase in activity of NF-κB; this was prevented by pre-treatment with anti-RAGE antibodies [54]. The authors suggested that inflammation and oxidative stress markers were possibly interrelated in end-stage renal diseases.

In addition to measurements of AGE products and immunohistochemical detection of RAGE in human diseased and normal tissue sections, other reports examine the relationship between soluble levels of RAGE in human plasma/serum and pathological disorders. Two forms of soluble RAGEs have been detected in human plasma/serum; the first is cell surface-cleaved soluble RAGE [55,56] and the second form of soluble RAGE, is termed endogenous secretory RAGE. In contrast to cell surface cleaved RAGE, esRAGE is produced via an alternative splicing mechanism [57,58]. In general, esRAGE is believed to represent approximately 20% of the total sRAGE content.

Santilli and colleagues studied total plasma sRAGE levels, urinary 8-iso-prostaglandin F (PGF) 2α excretion in urine and plasma levels of asymmetric dimethylarginine (ADMA) in 60 hypercholesterolemic subjects and 20 controls. They reported that plasma sRAGE was significantly lower and ADMA and urinary 8-iso-PGF2α were higher in the group with hypercholesterolemia *vs*. controls. Multivariate regression analyses revealed that only 8-iso-PGF2α and ADMA predicted the sRAGE levels [59]. In subjects with hypercholesterolemia, treatment for 8 weeks with statin therapy (pravastatin or atorvastatin) resulted in a significant reduction in levels of urinary 8-iso-PGF2α and treatment with atorvastatin resulted in a rise in sRAGE levels close to those in the normal controls; no changes in ADMA were noted. The authors concluded that sRAGE might be a protective factor against oxidative stress and endothelial dysfunction in atherosclerosis. Of note, however, the study did not distinguish total sRAGE *vs*. esRAGE components in the experiments.

Experimental evidence has strongly linked RAGE and its ligands to oxidative stress in diabetes [3,13]. Devangelio and colleagues tested these concepts in human subjects. They examined levels of sRAGE, ADMA and urinary 8-iso-PGF2α in 86 diabetic subjects and 43 controls. They reported that levels of plasma sRAGE were lower and levels of ADMA were higher in the diabetic *vs*. control subjects and that a measure of glycemic control, hemoglobin A1c (HbA1c) and urinary 8-iso-PGF2α were independently related to sRAGE levels in diabetic subjects. Of the 86 diabetic subjects, 24 with newly diagnosed diabetes and 12 of the subjects with poor metabolic control received therapies including hypoglycemic agents or insulin, respectively [60]. The authors reported that improvement in metabolic control by these agents resulted in a significant rise in sRAGE levels in parallel with reduction in ADMA levels. The authors concluded that oxidative stress and endothelial dysfunction characteristic of diabetes might be associated with lower sRAGE levels and that attempts to improve metabolic control might be marked by improvements (increases) in the levels of sRAGE.

Rodino-Janeiro and colleagues examined expression of RAGE and oxidative stress markers in human adipose tissue and the relationship to coronary artery disease [61]. They examined human subjects undergoing heart surgery and divided them into two groups, those with and without coronary artery disease. Biopsies of epicardial adipose tissue and subcutaneous adipose tissue were performed and revealed that the expression of RAGE mRNA transcripts and protein in the subcutaneous tissue form subjects with coronary artery disease was lower than that in subjects without coronary artery disease. No changes in RAGE expression were noted in the epicardial adipose tissue between these two groups of subjects. However, p22phox and RAGE gene expression were higher in the epicardial *vs*. the subcutaneous adipose tissue, in parallel with reduced levels of catalase mRNA. Interestingly, the subunits of NADPH oxidase were not impacted by the presence or absence of coronary artery disease. The authors concluded that a potential relationship between RAGE expression levels and ROS production in adipose tissue was possible.

### 6.2. Pulmonary Disease

Inghilleri and colleagues examined sections from lung tissue retrieved from subjects with usual interstitial pneumonia (UIP) *vs*. cryptogenic organizing pneumonia (COP) for levels of RAGE, oxidative stress, nitrosylation and fibroblast lesions [62]. They reported that in both forms of pneumonia, RAGE expression was equivalently high. However, levels of superoxide dismutases and iNOS, which were diffusely present in COP endoalveolar plugs, were not detected in UIP fibroblasts lesions. Tissue nitrosylation was also found to be lower in UIP *vs*. COP. The authors concluded that while RAGE expression was high in both settings, suppression of antioxidant enzyme expression was only observed in UIP. These data suggest that oxidative stress pathways may exert distinct influences in UIP *vs*. COP, both of which are characterized by increased expression of RAGE and fibroblast lesions. It is notable, however, that the authors did not study specific RAGE ligands—such as AGEs, S100/calgranulins or HMGB1 in this work; it is conceivable that insights into the varied findings might underlie their findings.

### 6.3. Central Nervous System

Chronic neurodegenerative disorders in human subjects are well-known for their association with oxidative stress. In the setting of diseases characterized by Lewy bodies, Dalfo and colleagues showed that compared to the brains of healthy matched controls, subjects with Lewy bodies displayed the following: increased mass spectrometric and immunological evidence of increased malondialdehyde lysine (MDAL) and 4-hydroxynonenal lysine (HNE), increased AGEs and heterogeneous expression of RAGE in multiple regions in the brain, such as substantial nigra, amygdala and frontal cortex [63]. Increased concentration of docosahexaenoic acid in the amygdala and frontal cortex was also demonstrated. These authors were able to further identify two proteins bearing oxidative damage, α-synuclein and manganese superoxide dismutase in incidental Lewy body disease cortex. Thus, by localizing AGE-RAGE to oxidative stress, the study supports that a mechanistic role for this pathway in the generation of ROS in Lewy body disease is plausible.

In a distinct study, Freixes and colleagues examined cerebral cortex in Creutzfeldt-Jakob disease. Immunohistochemistry studies were performed to test the concept that expression of oxidative, glycoxidative, lipoxidative and nitrative protein damage were evident in the cerebral cortex in this disorder. The authors found that expression of CML and carboxy ethyl lysine (CEL) AGEs, 4-hydroxynonenal, MDAL, nitrotyrosine, neuronal, endothelial and inducible NOS isoforms, RAGE and superoxide dismutase1 and manganese superoxide dismutase 2 levels were increased in Creutzfeldt-Jakob brain *vs*. healthy age-matched control brain [64]. They concluded that oxidative stress mechanisms might contribute to the pathophysiology of prion disease.

### 6.4. Immune/Inflammatory Disorders: Rheumatoid Arthritis

Rheumatoid arthritis (RA) is a chronic autoimmune disorder characterized by chronic inflammation leading to destruction of bones and joint tissues. Ferrante and colleagues examined markers of oxidative stress and plasma levels of esRAGE in 54 subjects with RA and 20 healthy control subjects [65]. The authors reported that levels of urinary markers of oxidative stress were higher in RA patients *vs*. controls and that levels of esRAGE were lower in RA subjects *vs*. controls. Interestingly, a direct correlation between the urinary markers of oxidative stress was observed only in patients not receiving anti-TNFα treatment. In contrast, subjects receiving anti-TNFα treatment displayed significantly lower levels of urinary 8-iso-PGF2α but not urinary 11-dehydro-thromboxane B2 (TXB) 2 *vs*. control subjects. In the latter case, esRAGE was the only independent predictor of the levels of urinary 11-dehydro-TXB2 [65]. The authors concluded that the anti-TNFα agents might only exert efficacy against isoprostane generation with no impact on thromboxane synthesis. Further, they posited that hyperactivity of the RAGE pathway might escape TNFα blockade, thereby contributing to the synthesis of thromboxane in these subjects.

### 6.5. Disorders of Reproduction: Preeclampsia

Preeclampsia, a complication of pregnancy, is associated with oxidative stress. Chekir and colleagues tested the hypothesis that AGE-RAGE and oxidative stress measures might differ in the placentas of human subjects with or free of preeclampsia. Serum levels of AGEs were found to be higher in subjects with preeclampsia compared to those with pregnancy but without preeclampsia and compared to normal healthy subjects [66]. Western blotting performed on the extracts of placental tissues revealed that levels of AGEs and RAGE were higher in women with preeclampsia *vs*. normal pregnancy. By immunohistochemical analyses, levels of 4-hydroxynonenal and 8-hydroxy-2′-deoxyguanosine were higher in the preeclamptic *vs*. normal pregnancy placenta [66]. These studies suggested possible mechanistic links between AGE-RAGE and the oxidative stress observed in the placental tissue in preeclampsia.

## 7. Summary and Perspectives

**Figure 1:**
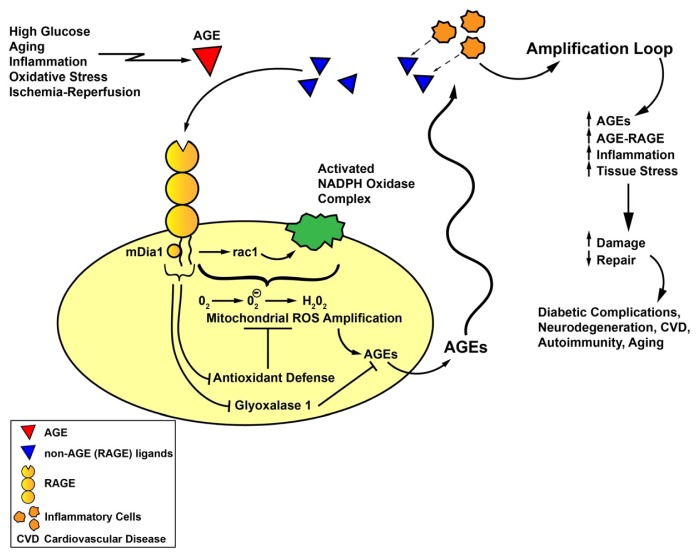
AGE-RAGE, oxidative stress and amplification loops linked to the pathogenesis of chronic disease. We propose that generation and accumulation of AGEs (**red triangles**) may be an important triggering event in a diverse array of stimuli, such as hyperglycemia, natural aging, inflammatory mechanisms and oxidative stress (such as via the myeloperoxidase pathway), and ischemia-reperfusion. Once AGEs achieve degrees of modification/concentrations sufficient to bind to and activate RAGE, RAGE signaling, at least in part through the interaction of its cytoplasmic domain with the formin, mDia1, results in activation of NADPH oxidase and the generation of ROS. Such ROS, via mitochondrial amplification, may generate even further ROS. One consequence of increased NADPH oxidase- and mitochondrial ROS generation is the consumption of antioxidant defenses. Together with RAGE-dependent suppression of Glo1, AGE formation and accumulation is sustained and amplified. Once set in motion, AGEs may stimulate recruitment of RAGE-expressing inflammatory cells which, when activated, may result in the release of non-AGE RAGE ligands, such as S100/calgranulins and HMGB1 (**blue triangles**). Such non-AGE + AGE ligands of RAGE may stimulate yet an additional amplification loop of RAGE activation, that is, cellular stress and activation of gene programs that augur tissue damage and reduced activation of repair mechanisms. We posit that such cellular stimulation mediated by AGE-RAGE and the indicated amplification loops contribute to the pathogenesis of the complications of diabetes, cardiovascular disease, autoimmunity and innate aging. Blocking AGE formation, RAGE signaling and the indicated amplification loops may be essential for the prevention of these chronic disorders.

Taken together, these considerations support a compelling multi-step role for ligand-RAGE interaction in diverse cell types and systems *in vivo* (both in animal models and in human subjects) in the pathogenesis of oxidative stress (Figure 1). Although oxidative stress has been proposed to contribute to a wide range of disorders, from aging, neurodegeneration, diabetes and its complications, autoimmunity, cardiovascular diseases, pulmonary disorders, and reproductive disorders such as preeclampsia, common threads linking increased generation of ROS and/or reduced antioxidative potential to precise pathogenic mechanisms have yet to be firmly demonstrated. We thus propose a model implicating AGE formation as an initial inciting event in the diverse array of disorders indicated above. We predict that AGEs, once formed, contribute to attraction and activation of inflammatory cells, or, in other settings, activation of endogenous cells—one consequence of which in both situations is the release/expression of non-AGE RAGE ligands, such as S100/calgranulins, HMGB1 or others. Such release and availability of these RAGE ligands may further amplify RAGE-dependent oxidative stress. Furthermore, as illustrated by enlightening experiments in UCP2 null mice discussed above, oxidative stress may beget further AGE production [47], thereby fueling the engines of oxidative and cellular stress and tissue damage. Additional evidence supporting this premise is deduced from the work of Anderson and Heinecke in which they showed that phagocyte-derived oxidants from NADPH oxidase are required for the production of CML AGEs during inflammation [67]. Together with RAGE-dependent suppression of Glo1 (as identified in diabetic renal cortical tissue) [68], these considerations suggest that RAGE might be a key nodal point in generation of ROS and the pathogenesis of chronic disease. Studies are underway to unravel the precise roles of mDia1 in RAGE-dependent oxidative stress to determine if RAGE signal transduction itself is a logical and effective target for therapeutic intervention in the diverse array of RAGE-associated pathologies characterized by accelerated oxidative stress.

## References

[1] Schmidt A.M., Vianna M., Gerlach M., Brett J., Ryan J., Kao J., Esposito C., Hegarty H., Hurley W., Clauss M. (1992). Isolation and characterization of two binding proteins for advanced glycosylation end products from bovine lung, which are present on the endothelial cell surface. J. Biol. Chem.

[2] Brett J., Schmidt A.M., Yan S.D., Zou Y.S., Weidman E., Pinsky D., Nowygrod R., Neeper M., Prysiecki C., Shaw A. (1993). Survey of the distribution of a newly characterized receptor for advanced glycation endproducts of tissues. Am. J. Pathol.

[3] Yan S.F., Ramasamy R., Schmidt A.M. (2010). The RAGE axis: A fundamental mechanism signaling danger to the vulnerable vasculature. Circ. Res.

[4] Hofmann M.A., Drury S., Fu C., Qu W., Taguchi A., Lu Y., Avila C., Kambham N., Bierhaus A., Nawroth P. (1999). RAGE mediates a novel proinflammatory axis: A central cell surface receptor for S100/calgranulin polypeptides. Cell.

[5] Taguchi A., Blood D.C., del Toro G., Canet A., Lee D.C., Qu W., Tanji N., Lu Y., Lalla E., Fu C. (2000). Blockade of RAGE-amphoterin signaling suppresses tumor growth and metastases. Nature.

[6] Yan S.D., Chen X., Fu J., Chen M., Zhu H., Roher A., Slattery T., Zhao L., Nagashima M., Morser J. (1996). RAGE and amyloid beta peptide neurotoxicity in Alzheimer’s disease. Nature.

[7] Rai V., Toure F., Chitayat S., Pei R., Song F., Li Q., Zhang J., Rosario R., Ramasamy R., Chazin W.J. (2012). Lysophosphatidic acid targets vascular and oncogenic pathways via RAGE signaling. J. Exp. Med.

[8] Chavakis T., Bierhaus A., Al-Fakhri N., Schneider D., Witte S., Linn T., Nagashima M., Morser J., Arnold B., Preissner K.T. (2003). The pattern recognition receptor (RAGE) is a counterreceptor for leukocyte integrins: A novel pathway for inflammatory cell recruitment. J. Exp. Med.

[9] Ma W., Rai V., Hudson B.I., Song F., Schmidt A.M., Barile G.R. (2012). RAGE binds C1q and enhances C1q-mediated phagocytosis. Cell. Immunol.

[10] Chang J.S., Wendt T., Qu W., Kong L., Zou Y.S., Schmidt A.M., Yan S.F. (2008). Oxygen deprivation triggers upregulation of early growth response 1 by the receptor for advanced glycation end products. Circ. Res.

[11] Sohal R.S., Allen R.G. (1990). Oxidative stress as a causal factor in differentiation and aging: A unifying hypothesis. Exp. Gerontol.

[12] Baynes J.W. (1991). Role of oxidative stress in development of complications in diabetes. Diabetes.

[13] Yan S.D., Schmidt A.M., Anderson G.M., Zhang J., Brett J., Zou Y.S., Pinsky D., Stern D (1994). Enhanced cellular oxidant stress by the interaction of advanced glycation end products with their receptors/binding proteins. J. Biol. Chem.

[14] Wautier M.P., Chappey O., Corda S., Stern D.M., Schmidt A.M., Wautier J.L. (2001). Activation of NADPH oxidase by AGE links oxidant stress to altered gene expression via RAGE. Am. J. Physiol. Endocrinol. Metab.

[15] Coughlan M.T., Thorburn D.R., Penfold S.A., Laskowski A., Harcourt B.E., Sourris K.C., Tan A.L.Y., Fukami K., Thallas-Bonke V., Nawroth P.P. (2009). RAGE-induced cytosolic ROS promote mitochondrial superoxide generation in diabetes. J. Am. Soc. Nephrol.

[16] Liu Y., Ma Y., Wang R., Xia C., Zhang R., Lian K., Luan R., Sun L., Yang L., Lau W.B. (2011). Advanced glycation endproducts accelerate ischemia-reperfusion injury through receptor for advanced end product/nitrative thioredoxin inactivation in cardiac microvascular endothelial cells. Antioxid. Redox Signal.

[17] Reddy M.A., Li S.L., Sahar S., Kim Y.S., Xu Z.G., Lanting L., Natarajan R (2006). Key role of Src kinase in S100B-induced activation of the receptor for advanced glycation endproducts in vascular smooth muscle cells. J. Biol. Chem.

[18] Gawdzik J., Mathew L., Kim G., Puri T.S., Hofmann Bowman M.A. (2011). Vascular remodeling and arterial calcification are directly mediated by S100A12 (EN-RAGE) in chronic kidney disease. Am. J. Nephrol.

[19] Omori K., Ohira T., Uchida Y., Ayilavarapu S., Batista E.L., Yagi M., Iwata T., Liu H., Hasturk H., Kantarci A. (2008). Priming of the neutrophil oxidative burst in diabetes requires preassembly of the NADPH oxidase. J. Leukoc. Biol.

[20] Suzuki Y., Inoue T., Yoshimaru T., Ra C (2008). Galectin-3 but not galectin-1 induces mast cell death by oxidative stress and mitochondrial permeability transition. Biochim. Biophys. Acta.

[21] Ruiter M.S., van Golde J.M., Schaper N.C., Stehouwer C.D., Huijberts M.S. (2010). Diabetes impairs arteriogenesis in the peripheral circulation: Review of molecular mechanisms. Clin. Sci. (Lond.).

[22] Felice F., Barsotti M.C., Poredos P., Balbarini A., di Stefano R (2013). Effect of aging on metabolic pathways in endothelial progenitor cells. Curr. Pharm. Des.

[23] Chen J., Huang L., Song M., Yu S., Gao P., Jing J (2009). *C*-reactive protein upregulates receptor for advanced glycation endproducts expression and alters defenses in rat endothelial progenitor cells. J. Cardiovasc. Pharmacol.

[24] Chen J., Song M., Yu S., Gao P., Yu Y., Wang H., Huang L (2010). Advanced glycation endproducts alter functions and promote apoptosis in endothelial progenitor cells through receptor for advanced glycation endproducts mediate overexpression of cell oxidant stress. Mol. Cell. Biochem.

[25] Du Yan S., Zhu H., Fu J., Yan S.F., Roher A., Tourtellotte W.W., Rajavashisth T., Chen X., Godman G.C., Stern D. (1997). Amyloid-beta peptide-receptor for advanced glycation endproduct interaction elicits neuronal expression of macrophage colony stimulating factor: A proinflammatory pathway in Alzheimer’s disease. Proc. Natl. Acad. Sci. USA.

[26] Nitti M., d’Abramo C., Traverso N., Verzola D., Garibotto G., Poggi A., Odetti P., Cottalasso D., Marinari U.M., Pronzato M.A. (2005). Central role of PKC delta in glycoxidation-dependent apoptosis of human neurons. Free Radic. Biol. Med.

[27] Wang Z., Li D.D., Liang Y.Y., Wang D.S., Cai N.S. (2002). Activation of astroyctes by advanced glycation endproducts: Cytokine induction and nitric oxide release. Acta Pharmacol. Sin.

[28] Vincent A.M., Perrone L., Sullivan K.A., Backus C., Sastry A.M., Lastoskie C., Feldman E.L. (2007). Receptor for advanced glycation endproducts activation injures primary sensory neurons via oxidative stress. Endocrinology.

[29] Qin J., Goswami R., Dawson S., Dawson G (2008). Expression of the receptor for advanced glycation endproducts in oligodendrocytes in response to oxidative stress. J. Neurosci. Res.

[30] Schurman L., McCarthy A.D., Sedlinsky C., Gangoiti M.V., Arnol V., Bruzzone L., Cortizo A.M. (2008). Metformin reverts deleterious effects of advanced glycation endproducts (AGEs) on osteoblastic cells. Exp. Clin. Endocrinol. Diabetes.

[31] Ide Y., Matsui T., Ishibashi Y., Takeuchi M., Yamagishi S (2010). Pigment epithelium derived factor inhibits advanced glycation endproduct elicited mesangial cell damage by blocking NF-κB activation. Microvasc. Res.

[32] Chen S.C., Guh J.Y., Hwang C.C., Chiou S.J., Lin T.D., Ko Y.M., Huang J.S., Yang Y.L., Chuang L.Y. (2010). Advanced glycation endproducts activate extracellular regulated kinase via the oxidative stress EGF receptor pathway in renal fibroblasts. J. Cell. Biochem.

[33] Lee B.W., Chae H.Y., Kwon S.J., Park S.Y., Ihm J., Ihm S.H. (2010). RAGE ligands induced apoptotic cell death of pancreatic-beta cells via oxidative stress. Int. J. Mol. Med.

[34] Sun L., Ishida T., Yasuda T., Kojima Y., Honjo T., Yamamoto Y., Yamamoto H., Ishibashi S., Hirata K., Hayashi Y (2009). RAGE mediates oxidized LDL induced pro-inflammatory effects and atherosclerosis in non-diabetic LDL receptor deficient mice. Cardiovasc. Res.

[35] Hofmann-Bowman M., Wilk J., Heydemann A., Kim G., Rehman J., Lodato J.A., Raman J., McNally E.M. (2010). S100A12 mediates aortic wall remodeling and aortic aneurysm. Circ. Res.

[36] Tikellis C., Thomas M.C.., Harcourt B.E., Coughlan M.T., Pete J., Bialkowsi K., Tan A., Bierhaus A., Cooper M.E., Forbes J.M. (2008). Cardiac inflammation associated with a Western diet is mediated via activation of RAGE by AGEs. Am. J. Physiol. Endocrinol. Metab.

[37] Bucciarelli L.G., Kaneko M., Ananthakrishnan R., Harja E., Lee L.K., Hwang Y., Lerner S., Bakr S., Li Q., Lu Y. (2006). Receptor for advanced glycation endproducts: Key modulator of myocardial ischemic injury. Circulation.

[38] Bucciarelli L.G., Ananthakrishnan R., Hwang Y.C., Kaneko M., Song F., Sell D.R., Strauch C., Monnier V.M., Yan S.F., Schmidt A.M. (2008). RAGE and modulation of ischemic injury in the diabetic myocardium. Diabetes.

[39] Loor G., Kondapalli J., Iwase H., Chandel N.S., Waypa G.B., Guzy R.D., vanden Hoek T.L., Schumacker P.T. (2011). Mitochondrial oxidative stress triggers cell death in simulated ischemia-reperfusion. Biochim. Biophys. Acta.

[40] Di Lisa F., Menabo R., Canton M., Barile M., Bernardi P (2001). Opening of the mitochondrial permeability transition pore causes depletion of mitochondrial and cytosolic NAD^+^ and is a causative event in the death of myocytes in postischemic reperfusion of the heart. J. Biol. Chem.

[41] Aleshin A., Ananthakrishnan R., Li Q., Rosario R., Lu Y., Qu W., Song F., Bakr S., Szabolcs M., D’Agati V. (2008). RAGE modulates myocardial injury consequent to LAD infarction via impact on JNK and STAT signaling in a murine model. Am. J. Physiol. Heart Circ. Physiol.

[42] Tsoporis J.N., Izhar S., Leong-Poi H., Desjardins J.F., Huttunen H.J., Parker T.G. (2010). S100B interaction with receptor for advanced glycation endproducts (RAGE): A novel receptor-mediated mechanism for myocyte apoptosis postinfarction. Circ. Res.

[43] Inagi R., Yamamoto Y., Nangaku M., Usuda N., Okamato H., Kurokawa K., van ypersele de Strihou C., Yamamoto H., Miyata T (2006). A severe diabetic nephropathy model with early development of nodule-like lesions induced by megsin overexpression in RAGE/iNOS transgenic mice. Diabetes.

[44] Tomino Y., Hagiwara S., Gohda T (2011). AGE-RAGE interaction and oxidative stress in obesity related renal dysfunction. Kidney Int.

[45] Guo J., Ananthakrishnan R., Qu W., Lu Y., Reiniger N., Zeng S., Ma W., Rosario R., Yan S.F., Ramasamy R. (2008). RAGE mediates podocyte injury in adriamycin-induced glomerulosclerosis. J. Am. Soc. Nephrol.

[46] De Oliveira M.R., Oliveira M.W., Behr G.A., de Bittencourt Pasquali M.A., Moreira J.C. (2009). Increased receptor for advanced glycation endproducts immunocontent in the cerebral cortex of vitamin A-treated rats. Neurochem. Res.

[47] Kuhla A., Hettwer C., Menger M.D., Vollmar B (2010). Oxidative stress associated rise of hepatic protein glycation increases inflammatory liver injury in uncoupling protein 2 deficient mice. Lab. Invest.

[48] Rabbani N., Thornalley P.J. (2011). Glyoxalase in diabetes, obesity and related disorders. Semin. Cell Dev. Biol.

[49] Ekong U., Zeng S., Dun H., Feirt N., Guo J., Ippagunta N., Guarrera J.V., Lu Y., Weinberg A., Qu W. (2006). Blockade of the receptor for advanced glycation endproducts attenuates acetaminophen toxicity in mice. Gastroenterol. Hepatol.

[50] Hudson B.I., Kalea A.Z., del Mar Arriero M., Harja E., Boulanger E., D’Agati V., Schmidt A.M. (2008). Interaction of the RAGE cytoplasmic domain with diaphanous1 is required for ligand-stimulated cellular migration through activation of Rac1 and Cdc42. J. Biol. Chem.

[51] Xu Y., Toure F., Qu W., Lin L., Song F., Shen X., Rosario R., Garcia J., Schmidt A.M., Yan S.F. (2010). Advanced glycation endproduct (AGE) receptor for AGE (RAGE) signaling and upregulation of Egr-1 in hypoxic macrophages. J. Biol. Chem.

[52] Toure F., Fritz G., Li Q., Rai V., Daffu G., Zou Y.S., Rosario R., Ramasamy R., Alberts A.S., Yan S.F. (2012). Formin mDia1 mediates vascular remodeling via integration of oxidative and signal transduction pathways. Circ. Res.

[53] Young K.G., Copeland J.W. (2010). Formins and cell signaling. Biochim. Biophys. Acta.

[54] Rodriguez-Ayala E., Anderstam B., Suliman M.E., Seeberger A., Heimburger O., Lindholm B., Stenvinkel P (2005). Enhanced RAGE-mediated NFKB stimulation in inflamed hemodialysis patients. Atherosclerosis.

[55] Raucci A., Cugusi S., Antonelli A., Barabino S.M., Monti L., Bierhaus A., Reiss K., Saftig P., Bianchi M.E. (2008). A soluble form of the receptor for advanced glycation endproducts (RAGE) is produced by proteolytic cleavage of the membrane-bound form by the sheddase a disintegrin and metalloprotease 10 (ADAM10). FASEB J.

[56] Zhang L., Bukulin M., Kojro E., Roth A., Metz V.V., Fahrenholz F., Nawroth P.P., Bierhaus A., Postina R (2008). Receptor for advanced glycation endproducts is subjected to protein ectodomain shedding by metalloproteinases. J. Biol. Chem.

[57] Park I.H., Yeon S.I., Youn J.H., Choi J.E., Sasaki N., Choi I.H., Shin J.S. (2004). Expression of a novel secreted splice variant of the receptor for advanced glycation end products (RAGE) in human brain astrocytes and peripheral blood mononuclear cells. Mol. Immunol.

[58] Cheng C., Tsuneyama K., Kominami R., Shinohara H., Sakurai S., Yonekura H., Watanabe T., Takano Y., Yamamoto H., Yamamoto Y (2005). Expression profiling of endogenous secretory receptor for advanced glycation endproducts in human organs. Mod. Pathol.

[59] Santilli F., Bucciarelli L., Noto D., Cefalu A.B., Davi V., Ferrante E., Pettinella C., Averna M.R., Ciabattoni G., Davi G (2007). Decreased plasma soluble RAGE in patients with hypercholesterolemia: Effects of statins. Free Radic. Biol. Med.

[60] Devangelio E., Santilli F., Formoso G., Ferroni P., Bucciarelli L., Michetti N., Clissa C., Ciabattoni G., Consoli A., Davi G (2007). Soluble RAGE in type 2 diabetes: Association with oxidative stress. Free Radic. Biol. Med.

[61] Rodino-Janeiro B.K., Salgado-Somoza A., Teijeira-Fernandez E., Gonzalez-Juanatey J.R., Alvarez E., Eiras S (2011). Receptor for advanced glycation endproducts expression in subcutaneous adipose tissue is related to coronary artery disease. Eur. J. Endocrinol.

[62] Inghilleri S., Morbini P., Campo I., Zorzetto M., Oggionni T., Pozzi E., Luisetti M (2011). Factors influencing oxidative imbalance in pulmonary fibrosis: An immunohistochemical study. Pulm. Med.

[63] Dalfo E., Portero-Otin M., Ayala V., Martinez A., Pamplona R., Ferrer I (2005). Evidence of oxidative stress in the neocortex in incidental Lewy body disease. J. Neuropathol. Exp. Neurol.

[64] Freixes M., Rodriguez A., Dalfo E., Ferrer I (2006). Oxidation, glycoxidation, lipoxidation, nitration and responses to oxidative stress in the cerebral cortex in Creutzfeld-Jakob disease. Neurobiol. Aging.

[65] Ferrante E., Vazzana N., Santilli F., di Cicco M., Lauriti C., di Battista L., Ciabattoni G., di Matteo L., Davi G (2010). Determinants of thromboxane biosynthesis in rheumatoid arthritis: Role of RAGE and oxidant stress. Free Radic. Biol. Med.

[66] Chekir C., Nakatsuka M., Noguchi S., Konishi H., Kamada Y., Sasaki A., Hao L., Hiramatsu Y (2006). Accumulation of advanced glycation endproducts in women with preeclampsia: Possible involvement of placental oxidative stress and nitrative stress. Placenta.

[67] Anderson M.M., Heinecke J.W. (2003). Production of Nepsilon-(carboxymethyl)lysine is impaired in mice deficient in NADPH oxidase: A role for phagocyte derived oxidants in the formation of advanced glycation endproducts during inflammation. Diabetes.

[68] Reiniger N., Lau K., McCalla D., Eby B., Cheng B., Lu Y., Qu W., Quadri N., Ananthakrishnan R., Furmansky M. (2010). Deletion of the receptor for advanced glycation endproducts reduces glomerulosclerosis and preserves renal function in the diabetic OVE26 mice. Diabetes.

